# Editorial: The cultural psychology of the COVID-19 pandemic

**DOI:** 10.3389/fpsyg.2023.1152889

**Published:** 2023-03-07

**Authors:** Glenn Adams, Markus Kemmelmeier, Yulia Chentsova Dutton, Lucian Gideon Conway

**Affiliations:** ^1^Department of Psychology and Kansas African Studies Center, University of Kansas, Lawrence, KS, United States; ^2^Interdisciplinary Social Psychology Ph.D. Program, University of Nevada, Reno, Reno, NV, United States; ^3^Department of Psychology, Georgetown University, Washington, DC, United States; ^4^Department of Psychology, Grove City College, Grove City, PA, United States

**Keywords:** COVID-19, cultural psychology, health, illness, mobility

## 1. Introduction

As people around the world settled into public health lockdown at the beginning of the COVID-19 pandemic, researchers in cultural psychology joined colleagues from a wide variety of academic disciplines in turning their attention to this global health emergency. Although COVID-19 is clearly a biological disease resulting from viral infection that wreaks havoc through physiological processes, the resulting pandemic was also the product of cultural-psychological processes. We edited this Research Topic (RT) on the *Cultural Psychology of COVID-19* to provide an outlet for work that illuminates those processes.

Our call for papers was open between June 15 and December 31, 2020. We received 38 distinct submissions in response to the call, of which 20 (52.63%) proceeded to publication, a rate that is roughly equal to that of the Cultural Psychology specialty of *Frontiers in Psychology* (*FCP*) as a whole (51.61%). Before discussing the content of the articles, we discuss important features of the editorial process.

A primary goal of *FCP* is to encourage the participation of people from outside the WEIRD (i.e., Western, educated, industrial, rich, and democratic; Henrich et al., 2010) settings that disproportionately constitute the field of psychology. The mission is not only to decenter whitestream experience, but also to denaturalize the Eurocentric modern individualist tendencies that the field of psychology tends to regard as an almost natural standard. We therefore found it encouraging that our pool of submissions included papers from authors or with participants based in 28 countries (plus one submission that had 96 authors based in 20 different countries), including 18 submissions originating in academically marginalized settings (see Bou Zeineddine et al., 2022) of Eastern Europe, Africa, Asia, and Latin America. Regrettably, this encouraging pattern in distribution of submissions did not extend to the distribution of published papers, which consisted disproportionately of submissions with authors based primarily in WEIRD or Global North settings (*f* = 14 articles from 19 submissions for a publication rate of 73.7%) to the exclusion of submissions with authors based in Eastern Europe, China, or Global South settings (*f* = 6 articles from 18 submissions for a publication rate of 33.3%), χ^2^(1, *N* = 37) = 6.060, *p* = 0.014.

There are several possible explanations for this divergence in publication rates. One possibility, of course, is some sort of cultural bias. Another possibility, not exclusive of the first, comes from a pattern concerning journal of origin. Although *FCP* hosted the RT, authors could also submit papers to the RT *via* the Personality and Social Psychology (*PSP*) section of *Frontiers in Psychology* or the Public Mental Health sections of *Frontiers in Psychiatry* and *Frontiers in Public Health*. Most submissions (*f* = 31, 81.6%) were to *FCP*, of which we selected the majority for publication (*f* = 19, 61.3%). By contrast, we published only one (14.3%) of the seven submissions to the other three journals, and this was a submission to *PSP*.^1^ Notably for the discussion of the divergence in publication success rates as a function of geographic origin, we declined to publish the five submissions from Public Mental Health sections, all of which were based in settings—Bangladesh, China, Iran, Libya, and Poland—that are relatively marginalized in hegemonic global academia.

We interpret this pattern in terms of what one might understand as (a sort of) disciplinary cultural bias in publication criteria. A central criterion for acceptance of articles to *FCP* (and to this RT in particular) is conceptual or theoretical contribution. It is not sufficient to conduct a scale development or replication study outside WEIRD settings. In addition, the study must inform questions of theoretical interest in the field of cultural psychology. It was the conclusion of the editors and reviewers that the submissions we received from outside *Frontiers in Psychology* did not meet this criterion. This may well reflect the different mission and scope of those particular journals, which—quite appropriately—are oriented toward dissemination of public health knowledge regardless of theoretical contribution.

## 2. COVID-19 in the background: Exploring general questions in an interesting historical context

This contrast in mission and scope is perhaps most evident in several articles of the RT for which the historical context of COVID-19 was background for investigation of broader ideas about the cultural-ecological foundations of mind. These included three articles that compared responses of participants in Chinese and North American settings. First, Yang et al. investigated the hypothesis that needs for compensatory control would lead people in different cultural settings to ascribe blame for COVID-19 to targets—individual doctors in Chinese settings and medical systems in U.S. settings—that were relatively less important in local systems of meaning. Second, Yap et al. observed that tendencies toward dialecticism would lead people in Chinese settings to report greater state optimism and well-being in the face of the COVID-19 emergency than people in Euro-Canadian settings would report. Third, Ai et al. found that participants in Chinese and U.S. settings would diverge in their understandings of prosocial motivation—with a relative emphasis on social obligation versus personal desire, respectively—as an explanation for compliance with COVID-19 public health measures.

Other articles considered comparison across settings other than China and North America. Karl et al. compared the validity of the Health Belief Model (Rosenstock, 1974) across Italian and Romanian contexts as a tool for predicting COVID-19 protective behavior. Ting et al. compared religious expression, illness representations, and perceived stress across Buddhist, Christian, and Muslim communities in Malaysia. Glückstad et al. compared mean levels of anxiety about spread of infectious disease *via* tourism and its relationships with predictors (e.g., COVID-19 knowledge and attitudes toward pleasure-seeking *via* the experience economy) across four countries—Japan, China, Denmark, and Italy—that varied along dimensions of region (East Asia and Europe) and pandemic severity.

Besides comparison across cultural settings, several articles used the COVID-19 pandemic as an opportunity to investigate variation in psychological processes as a function of variation in cultural engagement *via* scores on scale or demographic measures. For example, Li et al. conducted a study with Chinese participants in which they observed a positive relationship between reciprocal filial piety (but not authoritarian filial piety) and mental health. In a study of Romanian couples, Turliuc and Candel investigated socioeconomic and gender variation in the relationship between marital stress and satisfaction. Finally, Shekriladze et al. conducted a study of Georgian adults to investigate the relationship between personal-level individualism and collectivism and tendencies to engage in rational or affective coping.

## 3. COVID-19 on center stage: Cultural-psychological foundations of risk and public health compliance

In contrast to these examples, which tested questions of broader theoretical interest with COVID-19 as background, many RT articles examined outcomes directly related to the pandemic. In the sole article that used experimental methods, Miyajima and Murakami investigated the effect of message framing on the intention of Japanese participants to engage in prevention behavior. In contrast to an earlier study in a U.S. setting (Jordan et al., 2021), they observed no evidence that prosocial framing elicited greater prevention intention than did self-interest framing.

A more common focus was the relationship between measures of cultural-ecological engagement and COVID-related outcomes. For example, Xiao observed in a sample of Chinese university students that individual-level endorsement of vertical collectivism and horizontal individualism was positively related, but endorsement of vertical individualism was negatively related, with willingness to comply with COVID-19 public health mandates. Focusing on country-level indicators from 73 countries, Erman and Medeiros reported that cultural-psychological variables of uncertainty avoidance and long-term temporal orientation (Hofstede, 2010) positively predicted various measures of COVID severity during the first months of the pandemic. Similarly, Güss and Tuason examined country-level indicators from 76 countries and observed that higher rates of COVID-related death were associated with cultural-psychological variables of individualism (Hofstede, 2010) and egalitarian values (Schwartz, 2020). Kemmelmeier and Jami put together these two approaches by using multi-level modeling with both U.S. state-level collective indicators and individual beliefs as predictors of engagement with a cultural object: protective masks. Their analysis confirms the extent to which the act of wearing (or not) these objects is a cultural behavior rooted in collective beliefs about their efficacy and meaning.

Finally, two articles considered the implications of country-level variables on spatial mobility, a behavioral indicator of risk to COVID exposure and failure to comply with public-health guidelines. Atalay and Solmazer observed in data from 75 countries that scores on the value orientation of hierarchy (Schwartz, 2008) were positively associated with reductions in spatial mobility during the COVID-19 pandemic. Based on data from 39 countries, Freeman and Schug hypothesized and observed that relational mobility—beliefs about the extent to which “environments provide [people] with opportunities to freely choose and exit relationships” (p. 1)—was somewhat ironically related to greater decreases in spatial mobility following the onset of COVID-19. Whereas the general conclusion that emerges across most of these studies is that greater openness, looseness, or opposition to hierarchy is associated with greater COVID-19 risk, this latter study deviates from the pattern by suggesting that greater openness or sense of freedom from constraint—in the sense of relational mobility—is associated with freedom to choose protective (e.g., stay-at-home) measures.

## 4. Toward a cultural psychology of body and health

In addition to these 16 empirical reports, the RT includes three *perspective* articles. Sumner and Kinsella drew upon qualitative analyses of interviews in the UK and Ireland in their discussion of solidarity appraisal—the belief that people in the community are doing their part by adhering to public health guidelines—and its role in the experience of burnout among frontline workers. Raab et al. make the provocative argument that strategic gamification of COVID-19 information—something that one might criticize as making light of a serious matter—may serve as an analogy that results in more successful public health messages. Adams et al. drew upon qualitative analyses of interviews with Ghanaian Christian leaders alongside theory and research on the cultural psychology of relationality to speculate on implications of pandemic innovations—especially the move to virtual format—for the construction and experience of sociality.

The sole review article in the RT Bayeh et al. not only provides an overview of relevant research 18 months into the pandemic, but also (and more important) provides a conceptual framework for organizing knowledge about cultural-psychological foundations of health and well-being that will remain relevant beyond the particular context of COVID-19. Its resounding message is that “although COVID-19 is clearly a biological disease tied to a specific virus, the culture–mind relation at the heart of cultural psychology is nonetheless essential to understanding the pandemic” (Bayeh et al., p. 1). We highly recommend this article (alongside others; e.g., Kitayama et al., 2022) to colleagues and instructors who wish to use work about the COVID-19 pandemic to illuminate the cultural-psychological shaping of health, illness, and bodily experience.

A conclusion that emerges from both the review article Bayeh et al. and contributions to the RT is one that speaks to an important goal of *FCP*—re-thinking Eurocentric modern individualist tendencies—to which we referred earlier. Cultural-psychological habits of openness, looseness, and pursuit of authentic individual strivings and personal growth may yield superior experience in cultural ecologies that afford freedom from constraint. However, research on the COVID-19 pandemic (e.g., Salvador et al., 2020) illuminates how these same tendencies can put people and societies at greater risk of bad outcomes in situations that demand coordinated action and subordination of individual desires to collective goals. Lest we imagine that such situations are an extreme exception, research from settings outside whitestream or WEIRD centers of academic power (and the looming threat of ecological catastrophe) suggest that such situations of constraint are a basic human condition.

## Author contributions

GA wrote the original draft of the article. MK, LC, and YC edited the original draft. All authors contributed to the article and approved the submitted version.

## Footnotes

^1^Although this difference in publication rates was statistically significant, χ^2^(1, *N* = 37) = 5.061, *p* = 0.024, one should interpret this test with appropriate caution given that 50% of the relevant cells of the 2 × 2 contingency table had expected frequencies < 5.
